# Study protocol for COVID-19 breakthrough infections and vaccine-induced immune response among a cohort of healthcare workers, Bangladesh

**DOI:** 10.1371/journal.pone.0316121

**Published:** 2024-12-31

**Authors:** Md. Zakiul Hassan, Ahamed Khairul Basher, Mohammed Ziaur Rahman, Taufiqur Rahman Bhuiyan, Fahmida Chowdhury, Md. Kamal Hossain, Aninda Rahman, Md. Nazmul Islam, Lindsey M. Duca, Susan Cornelia Kaydos-Daniels, Benjamin A. Dahl, Firdausi Qadri, Nancy Ortiz

**Affiliations:** 1 Infectious Disease Division, Programme for Emerging Infections, International Centre for Diarrhoeal Disease Research, Bangladesh (icddr,b), Dhaka, Bangladesh; 2 Communicable Disease Control, the Director General of Health Services, Ministry of Health and Family Welfare Government of Bangladesh, Dhaka, Bangladesh; 3 US Centers for Disease Control and Prevention, Atlanta, GA, United States of America; National Center for Global Health and Medicine, JAPAN

## Abstract

**Background:**

To optimize vaccination strategies, it is useful to detect breakthrough infections and assess vaccine effectiveness in programmatic use. Monitoring emerging SARS-CoV-2 variants and vaccine effectiveness against them is also essential to determine the most effective vaccine options. This study aims to monitor SARS-CoV-2 breakthrough infections, the emergence of new SARS-CoV-2 variants, and host immune response during the peri-infection period of COVID-19. The study will also assess the uptake of the COVID-19 vaccine booster doses, and associated barriers or motivations among healthcare workers (HCWs).

**Methods:**

Leveraging an existing HCW cohort in Bangladesh, HCWs will be enrolled from purposively selected health facilities from four different administrative divisions across Bangladesh. We captured cohort data on HCW’s demographic information, clinical information, COVID-19 illness, and exposure, and vaccination histories for COVID-19. However, no biological specimens were collected for testing during the first phase of the cohort. In the current study, we plan to follow enrolled HCWs biweekly for suspected COVID-19 illness and capture relevant data including illness outcomes. Respiratory swab samples from symptomatic and a subset of asymptomatic HCWs will be tested for SARS-CoV-2 by rRT-PCR and positive samples will undergo Sanger sequencing to identify the SARS-CoV-2 variants of concern (VOCs). We will also perform Whole Genome Sequencing on a subset of SARS-CoV-2 positive samples with low CT values (Ct ≤ 30) to identify emerging SARS-CoV-2 variants. To examine the antibody response, we will collect blood samples from the participants at 12-week intervals for one year. We will use the EUROIMMUN kit and will also perform in-house ELISA to assess host immune factors with Luminex platform.

**Discussion:**

This proposed study will generate useful data on COVID-19 breakthrough infection and the durability of anti-SARS-CoV-2 antibodies among HCWs following vaccination. The findings on booster vaccination intention and uptake will inform government COVID-19 vaccination strategies. Information on circulating and emerging strains of SARS-CoV-2 and vaccine performance against those strains will help understand population-level risks of COVID-19 infection. The study will generate data on facilitators and barriers to COVID-19 booster uptake among HCWs which can inform health communication messaging to improve booster acceptance in this population.

## Background

The novel coronavirus SARS-CoV-2 has spread to more than 200 countries [1], and as of 05 April 2023, SARS-CoV-2 infected over 762 million people globally causing more than 6.8 million deaths [2]. The impact of COVID-19 on healthcare workers (HCWs) has been substantial. A recent systematic review indicated that the adjusted pooled prevalence of real-time reverse-transcriptase polymerase-chain-reaction (rRT-PCR)-confirmed COVID-19 among HCWs was 11% (95% CI: 7 to 16%) [3]. The World Health Organization (WHO) has estimated that a minimum of 115,500 HCWs have lost their lives globally because of COVID-19 [4].

Throughout various phases of the pandemic, multiple COVID-19 virus variants have emerged, and some have become the dominant variant accounting for most new infections. The emergence of these variants of concern (VOCs) (Alpha, Beta, Gamma, and Delta) was responsible for new waves of infections across the entire world [5]. Compared to the previous dominant variant, the Delta variant was reported to have increased transmissibility, higher viral load [6], and high reinfection rates [5]. It became the globally dominant variant because of its ability to escape from natural immunity [7]. More recently, another new variant, Omicron (B.1.1.529), emerged and caused another wave of infections. WHO declared Omicron a VOC on November 26, 2021, because of its highly transmissible nature and risk of immune evasion [8].

The spread of VOCs with significantly higher transmissibility (e.g., Omicron and Delta variants) raised concerns about the ability of vaccines to sustainably control SARS-CoV-2. These concerns were exacerbated by the increasing number of cases reported among persons who had completed the primary series as the Delta variant spread globally, potentially signaling a decreased effectiveness for VOCs [9]. A preliminary study in the UK on the vaccine effectiveness of the BNT162b2 (Pfizer/BioNTech) and ChAdOx1 (AstraZeneca) vaccines against the new variant found a 6% reduction in effectiveness for the BNT162b2 vaccine and a 12% reduction for the ChAdOx1 vaccine [10].

Detection of breakthrough infection (“a vaccine breakthrough infection is defined as the detection of SARS-CoV-2 RNA or antigen in a respiratory specimen collected from a person ≥14 days after receipt of all recommended doses of an Food and Drug Administration (FDA)-authorized COVID-19 vaccine”) [11] is potentially beneficially in defining vaccine effectiveness in programmatic use, determining when and how to prioritize additional (booster) doses, among which groups, and which type of vaccines provide longer immunity. Monitoring circulating emerging SARS-CoV-2 variants and the effectiveness of vaccines against the emerging variants is also essential for deciding which type of vaccine to use. All the COVID-19 vaccine products elicit detectable antibodies in the early stages; however, the antibody levels wane over time [12]. Hence, data on the duration of protection by vaccination is necessary to monitor and assess the need for further vaccination for emerging variants.

SARS-CoV-2 influences host immune responses that can lead to acute respiratory distress syndrome (ARDS) [13]. Multiple reports suggest that hyperactivation of the humoral immune pathway (e.g., increased Interleukin-6) during the infection period contributes to the development of ARDS [14]. Hence, exploring the host immune responses is important to characterize breakthrough infections and severe COVID-19.

Globally, healthcare workers (HCWs) have been disproportionately affected by COVID-19 because of their occupational risk exposure. Studies have shown that HCWs have experienced a significantly higher rate of COVID-19 morbidity, nearly ten times higher compared to the general population [15]. Given to an inadequate supply of personal protective equipment (PPE) and lack of infection prevention and control (IPC) practices, the risk of COVID-19 infection among HCW was greater in low- and middle-income countries (LMICs) such as Bangladesh [16] as compared to higher income countries with more resources to maintain stricter adherence to Infection prevention and control (IPC) measures. According to WHO, since the first day of COVID-19 case detection in Bangladesh on 8 March 2020 to 27 November 2022 a total of 2,036,527 COVID-19 cases were confirmed by RT-PCR, GeneXpert, and Rapid Antigen tests, including 29,431 related deaths with a case fatality rate (CFR) of 1.45% [17]. Approximately 9,402 symptomatic HCWs were infected, and 186 died from COVID-19 during the first year of the pandemic in Bangladesh [18].

To protect front-line HCWs, the Government of Bangladesh initiated COVID-19 vaccination among HCWs on January 27, 2021, with Covishield (ChAdOx1-S). Subsequently, other vaccines, including Pfizer-BioNTech (BNT162b2), Moderna (mRNA-1273), Verocell (BBIBP-CorV), were introduced [19]. Healthcare workers (HCWs) were prioritized for COVID-19 vaccination at the outset of vaccine introduction, and as of April 30, 2023, vaccine coverage among this population stands at 91% for the primary series (received both 1^st^ and 2^nd^ dose of COVID-19 vaccine) [20]. However, the uptake of booster doses among HCWs remains low, with only 60% completing their third dose and a mere 2% completing their fourth dose (personal communication).

The Bangladesh government has been actively supporting the vaccination of healthcare workers, providing vaccines free of charge. This initiative has been made possible through a combination of vaccines obtained through the COVAX program and direct government purchases. Nevertheless, a question mark looms over the sustainability of this effort if annual vaccinations become necessary. Uncertainties persist regarding how the government will manage the procurement and distribution of vaccines in the years to come, given the evolving nature of the pandemic and the need to safeguard the healthcare workforce. Planning for long-term vaccination strategies remains a crucial consideration for the government’s healthcare policy.

In June 2021, icddr,b in collaboration with the Communicable Disease Control (CDC), Director General of Health Services (DGHS), the Ministry of Health and Family Welfare, Government of Bangladesh, and with technical support from the US Centers for Disease Control and Prevention (US CDC) established a cohort of HCWs to prospectively record COVID-19 illness and assess the occupational risk of COVID-19 among HCWs. However, no biological samples were collected from the cohort participants during the first phase of the cohort primarily because of the funding support required for sample transportation and testing; however, rRT-PCR testing facilities were available at the health facilities and reported COVID-19 test results were recorded. With >90% HCWs receiving at least one dose of COVID-19 vaccine, this study intends to leverage the HCWs cohort platform to monitor COVID-19 breakthrough infections and the emergence of any new SARS-CoV-2 variants. We would also like to investigate immune response in COVID-19 peri-infection period among the HCWs by vaccine types and identify host immune responses for SARS-CoV-2 infection. Additionally, we propose to capture data on COVID-19 booster uptake among the cohort member, including assessing the barriers and motivations for booster uptake among HCWs.

## Methods

### Study setting

**Fig 1:**
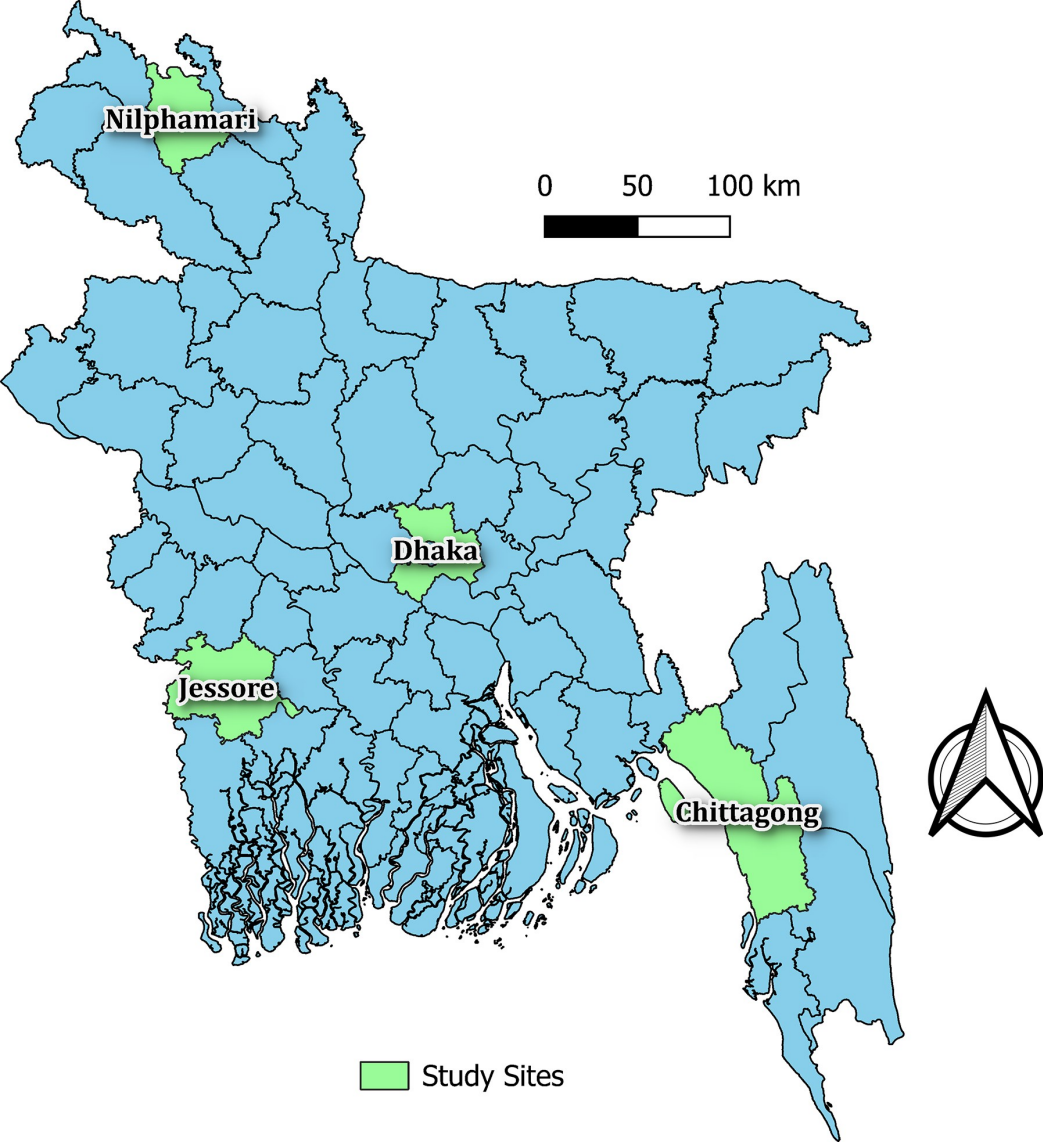
Location of the study facilities. *Source*: *Authors generated the map using QGIS version 3*.*2*.*1(**https*:*//qgis*.*org/**) and administrative-level open license shape files named “Bangladesh” from Global administrative areas (boundaries) GADM (**http*:*//gadm*.*org/country**)*.

We will leverage an established HCWs cohort platform. The cohort enrolled HCWs from purposively selected health facilities from four different administrative divisions across Bangladesh to establish a representative cohort of HCWs. All the HCWs aged above 18 years, and directly or indirectly involved in patient care were recruited from selected facilities. Spatial distribution of the study sites are demonstrated in Fig 1.

A summary of the name, type of facility, and number of beds in each facility is described in the tabulation format in Table 1.

**Table 1:** Name and type of healthcare facilities included in the cohort platform.

Sl.	Hospitals	District	Facility type/ care level	Administration	Beds	Patient population
1	Shaheed Suhrawardy Medical College hospital (SMCH)	Dhaka	Tertiary	Public	850	All age group
2	Holy Family Red Crescent Medical College Hospital	Dhaka	Tertiary	Private	646	All age group
3	Chittagong medical college hospital (CMCH)	Chittagong	Tertiary	Public	1500	All age group
4	USTC Bangabandhu Memorial Hospital	Chittagong	Tertiary	Private	220	All age group
5	Jessore district hospital	Jessore	Secondary	Public	250	All age group
	Seven upazila health complexes in Jessore	Jessore	Primary	Public	50	All age group
	Seventy Community clinics, UHC, Jessore	Jessore	Primary	Public	-	All age group
6	Nilphamari District Hospital	Nilphamari	Secondary	Public	250	All age group
	Five upazila health complexes in Nilphamari	Nilphamari	Primary	Public	50	All age group
	Fifty community clinics, UHC, Nilphamari	Nilphamari	Primary	Public	-	All age group

### Study design

This is a prospective cohort study. Participants’ sociodemographic and epidemiological data were collected at enrolment. The enrolled participants will be prospectively followed-up every two weeks for symptom screening and to update their vaccination status.

### Participants enrolment in cohort

For this cohort, the HCW was defined as all staff in the healthcare facility involved in the provision of care to patients, including those who may not have provided direct care to the patient but who may have had contact with the patient’s body fluids, potentially contaminated items or environmental surfaces. This includes healthcare professionals, allied health workers, auxiliary health workers such as cleaning and laundry personnel, radiology physicians and technicians, clerks, phlebotomists, respiratory therapists, nutritionists, social workers, physical therapists, laboratory personnel, and cleaners, patient transporters, catering staff etc. The cohort excluded HCWs of basic medical science faculty or administrative staff who are not involved in providing clinical care.

### Baseline data collection for the cohort participants

Two field staff, a study physician, and a study nurse, have been deployed as full-time icddr,b staff in each district to recruit HCWs from all the participating healthcare facilities in that district. All HCWs in the study facility are approached, and the study physicians/nurses enrol those who are willing to participate and provide written informed consent in the presence of a witness. The participant and witness both carefully read the terms and details of the study and signed the consent form for record. The physician or nurse (clinician) then interviews the HCWs face-to-face to collect baseline data including socio-demography (e.g., age, sex, socioeconomic status), clinical information including pre-existing as well as current medical history, history of respiratory illness since the start of the epidemic in Bangladesh, history of a positive test for COVID-19, travel history, history of exposure to SARS-CoV-2 (e.g., exposure to COVID-19 patients, use of personal protective equipment, compliance with IPC measures, involvement in aerosol-generating procedures), COVID-19 vaccination status (date of vaccination, doses, vaccine brand), other vaccination histories (e.g., influenza and Hepatitis B vaccination), and any adverse events following COVID-19 vaccination using standardized questionnaire programmed in smart-phone (**S1 File**). Self-reported vaccination status is verified and confirmed by a vaccination card. The study clinician also carries out anthropometric measurements such as weight, height, waist circumference, blood pressure, and blood glucose level to determine co-morbidities [21]. The baseline data collection for the cohort was started in July 2021 and completed in the following year. However, no biological samples were collected from the cohort participants during the first phase. Routine bi-weekly follow-up started from the 14^th^ day of baseline enrolment and will be continued up to the study end date (31^st^ December 2024). During routine follow-up, the study team collected all records of COVID-19 tests that were performed by the participants when they had COVID-19 symptoms. The protocol described in this manuscript will be implemented during the second phase of the cohort. Data and sample collection activities in this phase started from 1^st^ January 2023 and planned to be ended on 31^st^ December 2024.

### Ethics approval and consent to participate

The institutional review board (IRB) of the International Centre for Diarrhoeal Disease Research, Bangladesh (https://www.icddrb.org/) reviewed and approved the study protocol (protocol reference number: PR-22113). This activity was reviewed by CDC and was conducted consistent with applicable federal law and CDC policy. We will obtain written informed consent from all study participants. The study team will provide detailed oral and written information in the local language (Bangla) on research aims, objectives, risks and benefits of participation, maintaining confidentiality, the right not to participate and/or withdraw conflicts of interest, and compensation. After being reminded that their participation is entirely voluntary and that any information they provide will be kept strictly confidential, participants will be asked to sign the consent form.

#### Investigation of breakthrough infections, immune response, and booster uptake

Cohort participants will be followed prospectively to identify suspected COVID-19 cases and collect nasopharyngeal swabs and serum specimens to confirm COVID-19 breakthrough infections. Additionally, we will track the emergence of any new SARS-CoV-2 variants and investigate immune response in COVID-19 peri-infection period among the HCWs by vaccine product types and identify host immune responses for SARS-CoV-2 infection. We will also collect data on COVID-19 booster uptake among the HCWs, including assessing the barriers and motivations for booster uptake.

The study team will maintain a follow-up logbook for all enrolled cohort participants. The team will keep the phone contact numbers and home addresses for each participant. The study clinician will call the participants biweekly to check on their health status. During the study follow-up, the clinician will record information on any illness, outpatient visit, or hospitalization since the last follow-up; suspected COVID-19 symptoms, date of symptom onset, date of PCR testing, if any, and PCR results; changes in potential COVID-19 risk exposure (e.g., working in different wards or exposure to COVID-19 cases); change in COVID-19 vaccination status, number of doses including booster dose, and date of vaccination and brand; and any adverse events following vaccination. The study team will encourage the unvaccinated healthcare workers to receive COVID-19 vaccination and will provide any logistical support to access vaccine if required. The team will also provide the list of unvaccinated HCWs to the hospital authority for awareness and follow-up to ensure all HCWs are fully vaccinated.

To assess uptake of booster doses, defined as COVID-19 vaccine administered to a person four months since their last dose, who has already completed their primary vaccination series [22], we will use a previously validated tool that contains information on the status of COVID-19 vaccination, booster uptake status, date of the booster, future intention, access to booster doses, protection motivation theory (PMT) scale, and vaccine hesitancy (VH) Scale (**S1 File**). To investigate the immune status of HCWs, participants will be followed up for serum antibody testing every 12 weeks in addition to the routine biweekly follow-up visit.

### Sample collection

#### Respiratory swabs

The study physician/ nurse will collect respiratory swabs (nasopharyngeal and oropharyngeal swabs) from the HCWs meeting the WHOs suspected COVID-19 clinical case definition: “*Acute onset of fever AND cough; OR Acute onset of ANY THREE OR MORE of the following signs or symptoms*: *fever*, *cough*, *general weakness/fatigue*, *headache*, *myalgia*, *sore throat*, *coryza*, *dyspnoea*, *anorexia/nausea/vomiting*, *and diarrhoea*.” [23]

We will also collect respiratory swabs biweekly from a subset of asymptomatic HCWs. We will stratify all the HCWs in different strata proportionate to the number (e.g., doctors, nurses, and support staff) and randomly select a subset of the asymptomatic HCW for respiratory swabs collection (around 50 per month, which can be supported within the study budget). The test results will be shared with the participants as soon as it is available. Our field staff will also notify the hospital focal person for the study and the hospital administration of necessary actions [Table 2].

**Table 2:** Schedule of baseline and follow-up data and sample collection.

Timing in the study	At enrolment	Biweekly	Suspected symptomatic COVID-19 cases and a subset of asymptomatic participants	Every twelve weeks
Baseline questionnaire	X			
Follow-up questionnaire including information on booster doses		X		
rRT-PCR test			X	
Serology	X			X

#### Blood specimens

To examine the antibody response to different vaccine doses and brands, we will collect blood specimens from a random subset of HCWs (a sub-cohort of approximately 1000 HCWs) at enrolment and every 12 weeks for one year. The trained study nurse deployed at the participating healthcare facility will collect blood specimens. The staff collecting and transporting the specimens will be trained in safe handling practices and spill decontamination procedures. Taking all aseptic precautions, the study nurse will collect 10 ml of blood from the mid-cubital vein using a butterfly needle, vacutainer and plasma tube. The study nurse will run the centrifuge to separate plasma from the blood specimen. Using pipettes or droppers, the study nurse will transfer the plasma from the plasma tube to the container. If not shipped daily, then the plasma will be stored inside the -20° c chamber of the freezer located at the field site (**Table 2**).

### Sample processing, storage and transportation

For each sample collected, the time of collection, the conditions for transportation and the time of arrival at the study laboratory will be recorded. From the study sites located in Dhaka, the specimens will be transported daily to icddr,b laboratory. For daily transport, the specimens will be shipped inside cool box with ice packs at 4–8° C. At the study sites located outside Dhaka, the specimens will be stored temporarily at -20° c chamber of the freezer and will be transported from study sites to icddr,b’s laboratory on a biweekly basis. The specimens will be transported to Dhaka, maintaining cold chain.

The specimens will be stored at -80° C or lower in the icddr,b’s laboratory. Every effort should be given to prevent repeated freezing and thawing of the specimens. The specimens will be aliquoted prior to freezing to minimize freeze-thaw cycles. Also, we will try to reduce the duration of storage in the freezers located at the study sites owing to their wide temperature fluctuations.

### Sample testing

#### Respiratory swabs

icddr,b laboratory will test the swab samples. Viral nucleic acid will be extracted using InviMag Universal kit (Invitek, STRATEC Molecular, Berlin-Buch, Germany) on Kingfisher Flex 96 (Thermo Fisher Scientific Inc.) automated nucleic acid extraction system according to the manufacturer’s instructions. The respiratory (nasopharyngeal and nasal) swab will be tested for SARS-CoV-2 by real-time reverse transcriptase PCR using RdRp (open reading frame 1ab [ORF1ab]) and N gene-specific primers and probes (China CDC).

The real-time RT-PCR positive samples will be subjected to two-step conventional rRT-PCR by using the High-Capacity cDNA Reverse Transcription Kit (Applied Biosystems, Foster City, CA, USA) for reverse transcription to generate cDNA following the manufacturer’s protocol. SARS-CoV-2 Spike (S) gene will be amplified by a specific primer set (ARTIC Primer set) using Qiagen HotstarTaq Polymerase Kit (Qiagen, Hilden, Germany) to detect different SARS-CoV-2 VOCs. All PCR products will be analysed by agarose gel electrophoresis with SYBR Safe staining (Thermo Fisher Scientific) and purified using the ExoSAP-IT kit (Affymetrix, OH, USA). Sanger sequencing will be carried out in the ABI 3500 XL genetic analyser (Applied Biosystems, Foster City, USA) using the forward and reverse primers separately.

Whole Genome sequencing will be conducted with a subset of samples selected based on the CT values (≤ 30) of real-time RT-PCR assays. On average, if 10%-20% of the total cases were positive for SARS-CoV-2 RNA PCR, we are expecting 20% of the positive samples will be of Ct ≤ 30. Thus, a subset of 104 to 140 samples will be subjected to Whole Genome sequencing by the Oxford Nanopore MinION platform. Moreover, samples will be tested in a batch of 48 or 96 samples at a time to minimize the cost.

Complementary DNA will be obtained using the LunaScript® RT SuperMix Kit (New England Biolabs®), and ARTIC nCoV-2019 V3 primer panel will be used to generate around 400 bp amplicons in two different PCR pools using Q5® Hot Start High-Fidelity 2× Master Mix. The Nanopore sequencing library will be prepared using a ligation sequencing kit (SQK-LSK109) and native barcode expansion packs EXP-NBD104 and EXP-NBD114 or EXP-NBD196. The library will be quantified by the Qubit dsDNA High Sensitivity Assay Kit (Invitrogen) with a Qubit fluorometer (Invitrogen). The final library will be sequenced on the FLO-MIN106D flow cell on an Oxford Nanopore MinION MK 1C platform with real-time base-calling. QC passed FASTQ reads will be analysed based on the ARTIC FieldBioinformatics pipeline. SARS-CoV-2 variant will be assigned based on Nextclade and Phylogenetic Assignment of Named Global Outbreak Lineages (Pangolin) software tools. It has been designed to conduct Whole Genome sequencing with samples selected based on the CT values (≤ 30) of real-time RT-PCR assays, which is a standard protocol for variant identification considering the frequent emergence of new variants and subvariants over time. We do not have the high containment biosafety facility to culture SARS-¬CoV-2, and not all samples will have CT values ≤ 30 in real-time RT-PCR. Under these circumstances, we planned to conduct S gene-specific sanger sequencing to increase the coverage of testing samples for screening circulatory SARS-CoV-2 variants. We believe that combining WGS and S gene-specific sequencing data will strengthen the methods to capture the emergence of any new SARS-CoV-2 variants.

#### Serum specimens

icddr,b immunology laboratory will test the serum specimens. The serum samples will be tested for IgG against SARS-CoV-2. We will use the EUROIMMUN kit (Lübeck, Germany; catalog number: EI2606-9601-10G) for antibody detection. In brief, diluted plasma will be incubated in reaction wells, each coated with recombinant SARS-CoV-2 spike protein. Specific IgG antibodies that bound to the spike protein antigen will be detected using an enzyme-conjugated colorimetric technique. The quantitative results will be calculated as a ratio of the extinction of colour of the control or tested specimen over the extinction of calibrators. The anti-S1 IgG antibody levels will be calculated using a standard curve generated from 6-point calibrators. We will consider antibody levels of ≥35.2 BAU/ml as positive. Serum samples will also be subjected to in-house ELISA for assessment of host immune factors (e.g., Interleukins, TNF-alpha, IFN-gamma, GM-CSF, etc.) depending on the scope of work, we may also deploy Luminex platform to perform the assay.

Antibody testing can be used for clinical, occupational health, and public health purposes, such as serologic surveys, to help differentiate past infection from vaccination by using tests that measure antibodies against different protein targets. The choice of antigen is also important for biological rather than technical reasons. For example, approximately 10% of PCR-positive individuals do not have detectable anti-nucleocapsid (N)-directed IgG responses (Weisberg SP, 2021, Nature Immunology), although they do have anti-S and anti-RBD responses at the same time. Therefore, by comparing the following antibody responses and data from clinical datasets, vaccine-induced and natural infection-induced antibody responses will be evaluated and analysed in the current study.

### Sample size estimation and sampling

#### Objective

Investigate immune response (IgG antibody and biomarkers level) following COVID-19 vaccination among the HCWs

To estimate the assumption for seroprevalence of anti-SARS-CoV-2 antibody among HCWs, we obtained prior estimates from a study by TR Bhuiyan and colleagues [24]. The study found an adjusted prevalence of SARS-CoV-2 antibodies of 64.1% among Bangladeshi residents in a rural subdistrict [24]. So, we expect that the seroprevalence will be P = 64% among the HCWs. Hence, we will require a sample of size, N = 355 HCWs, to estimate the expected proportion of 64% of the outcome within 5 percentage points, the absolute precision, and 95% confidence. Considering the 15% non-response rate, we will require at least 419 HCWs.

#### Objective

Assess the COVID-19 booster uptake among HCWs

To the best of our knowledge, there is no documented information on COVID-19 booster uptake in the Bangladeshi population. A study by Koh et al. estimated COVID-19 booster dose uptake and report that about 70% of eligible HCWs had taken COVID-19 booster dose (received at least 3^rd^ dose or received both 3^rd^ and 4^th^ dose of COVID-19 vaccine) [25].

We expect that the proportion of booster uptake will be P = 70% among the HCWs. We will require a sample of size, N = 323 HCWs to estimate the expected proportion 70% of the outcome within 5 percentage points, the absolute precision and 95% confidence levels. Considering 15% non-response rate, we will require at least 372 HCWs.

#### Objective

Monitor SARS-CoV-2 breakthrough *infections*

We consulted data from Bergwerk et al. where they documented that about 2% of the fully vaccinated HCWs developed SARS-CoV-2 breakthrough infections [26]. So, we expect that the breakthrough infection rate per year will be 2% among the HCWs. So, we will require a sample of size, N = 753 HCWs to estimate the expected proportion of 2% of the rate within 1 percentage points, the absolute precision and 95% confidence levels. Considering 15% dropout rate, we will require at least 866 HCWs.

Therefore, an estimated 866 HCWs will be enrolled in the study to meet the study objectives. However, as we plan to leverage an existing HCWs cohort, we plan to enrol all HCWs in this study (n = 3600) which will be sufficient to meet all study objectives (Table 3).

**Table 3:** Objective-specific sample size.

Sl.	Objectives	Sample size
1.	Investigate immune response following COVID-19 vaccination among the HCWs	419
2.	Assess the COVID-19 booster uptake among HCWs	372
3.	Monitor SARS-CoV-2 breakthrough infections	866

Since the beginning of the pandemic, we have observed some variations in the infection rate of SARS-CoV-2 at different times of the year. Despite the low infection rate at present, we plan to conduct the study for one year, which expectedly will provide us with desired sample size. We believe our study will capture data on circulating SARS-CoV-2 variants and COVID-19 booster uptake even if the circulation remains low throughout the year.

### Data analysis

#### Estimating the incidence of breakthrough infections

We will define COVID-19 breakthrough infection as ‘the detection of SARS-CoV-2 RNA or antigen in a respiratory specimen collected from a person ≥14 days after receipt of all recommended doses of an FDA-authorized COVID-19 vaccine [22, 26]’.

We will calculate the incidence of lab-confirmed SARS-CoV-2 by rRT-PCR among symptomatic HCWs using the following formula:

Incidence rate among symptomatic =

number of new cases of SARS-CoV-2 infection among symptomatic HCWs × 100

Total HCW-time at risk

#### Risk factors for breakthrough infection

We will calculate hazard ratios with 95% confidence intervals (95% CI) using Andersen-Gill extension of the Cox proportional hazards model to identify potential factors associated with breakthrough infections (outcome of interest). We will use the Cox proportional hazards model to analyze the outcome variable "Time to breakthrough infection." Additionally, we will incorporate time-varying covariates (e.g., comorbidities) into the model to identify potential risk factors that might influence the time to breakthrough infection. Follow-up will be from baseline to the earliest outcome or study exit. All potential confounders such as age, gender, vaccination status and type, comorbidities, and adherence to preventive measures will be adjusted in the multivariable Cox regression.

#### Immune status by vaccine brand

We will analyze IgG antibody response to different vaccine dose and vaccine brands combination: a) one dose of Covishield/ Pfizer-BioNTech/ Moderna (mRNA-1273)/ Verocell (BBIBP-CorV)/; b) two doses of homologous vaccine and c) two doses of homologous and one dose of heterologous vaccine. We will calculate the days from the last dose as duration between last vaccination and blood collection. We will assess differences in antibody levels between time periods using the Kruskal-Wallis (>2 groups), or the Mann–Whitney (two groups) test, as appropriate. We will calculate the median antibody levels from estimation of a fractional polynomial of days from the last dose and will plot the resulting curve along with the 95% confidence interval of the median. We will also analyze the proportion of SARS-CoV-2 infection, hospitalization, and death stratified by vaccine type and immune status.

#### Uptake of COVID-19 booster

We will calculate the proportion of HCWs receiving COVID-19 booster dosage and will stratify by vaccine brands, facility type, HCWs type, patient care role and basic demography. We will also assess intention and influencing factors regarding the booster dose uptake. We will use protection motivation theory (PMT) and vaccine hesitancy (VH) scale to calculate intention and factors associated with booster uptake. To ensure the reliability and validity of these scales, we will perform Cronbach’s α values and confirmatory factor analysis. We will then use the χ2 test to compare the proportions of participants with different COVID-19 booster vaccine intentions. The Wilcoxon rank-sum test will be used to compare PMT and VH scale scores between groups. We will also perform hierarchical multiple regression analyses to compare the predictive ability of the PMT scale and VH scale on booster vaccination intention. The hierarchical structure of our data includes individuals, wards, and department-level variables that will be employed in the hierarchical multiple regression analysis. This statistical technique allows us to model the outcome variable while accounting for the nested nature of the data. To determine if PMT constructs are associated with booster vaccination intention, multivariable logistic regression analyses will be conducted, adjusting for covariates. Odds ratios (OR) and 95% confidence intervals (CI) will be used to quantify the effects, with a significance level of p < 0.05.

## Data Safety Monitoring Plan (DSMP)

The study collects sociodemographic, and epidemiological data and information on test results from collected specimens. Using a unique identifying number, the data gathered from the participants will be kept confidential. The research team will guarantee the privacy of the data acquired from the participating HCWs, and all the information will be treated with strict confidentiality by masking the participant’s name or any other identifiable attributes and never be shared with outside parties. Additionally, only the principal investigator of the study will be the custody of data forms or any representative nominated by the principal investigator.

## Discussion

This prospective cohort study among a large cohort of HCWs in Bangladesh aims to monitor SARS-CoV-2 breakthrough infections and the emergence of new SARS-CoV-2 variants among this high-risk group. The study will also investigate immune response in COVID-19 peri-infection period and assess COVID-19 booster vaccine uptake, including barriers and motivations for vaccination among HCWs. These are critical data and would inform the country’s vaccination policy.

HCWs are undeniably the most vulnerable population for COVID-19 because of their direct exposure to infected patients [27]. COVID-19 infections among health workers are far greater than those in the general population [28]. Despite wider vaccination programme and prioritization among HCWs, breakthrough infections have been reported among this high-risk group [29]. Local data on prevalence and host immune responses of breakthrough infections remains limited among HCWs. To protect HCWs from repeat infections after vaccination, knowledge on their immune status and antibody kinetics is crucial. Our study will fill the knowledge gap.

In addition, this study also aims to monitor and whole sequence genetic factors of circulating and emerging SARS-CoV-2 variants. Many studies have indicated that genetic background plays an essential role in determining the host response to infections [30–32]. This study will provide such information on whether the existing vaccines are effective against the circulating and emerging variants as well as how long the vaccine provides immunity against the virus.

The World Health Organization (WHO) recommended COVID-19 booster dose vaccination 4–6 months following completion of the primary vaccination series for most at-risk populations such as HCWs [22]. Booster doses are necessary because of waning immunity and emerging variants [22]. Boosters have been proven to increase immunogenicity and peak antibody levels in healthy adults [33]. However, despite the WHO recommendation, booster vaccination uptake remains suboptimal in low and middle-income countries, including Bangladesh [34]. Acceptance of COVID-19 booster doses is influenced by several factors, such as fear of COVID-19 and trust in the vaccine. Our study will additionally investigate the reasons behind hesitancy toward COVID-19 booster doses. This will help us understand the factors that influence booster uptake and design effective vaccination campaigns and strategies.

While the study will inform policies for vaccination among high-risk populations such as HCWs the results may not be generalizable to other populations.

## Conclusion

This proposed study will provide valuable information on COVID-19 breakthrough infection and the durability of anti-SARS-CoV-2 antibodies among HCWs following vaccination program. The findings will inform COVID-19 vaccination strategies, including requirement and timing of booster doses. Information on circulating and emerging strains of SARS-CoV-2 and vaccine performance against those strains will help understand population-level risks of COVID-19 infection.

The prospective cohort design of our study is a major strength. Our cohort is comprised of a diverse range of health facilities, including public and private ones, and primary, secondary, and tertiary level facilities. Additionally, we included all types of front-line HCWs in our study, enabling the results to be more widely applicable and generalizable. We will share the key findings of our study with major stakeholders, including the participating hospitals, the Communicable Disease Control (CDC), the Director General of Health Services (DGHS), the Ministry of Health and Family Welfare of the Government of Bangladesh, the National Immunization Technical Advisory Group (NITAG), and the National COVID-19 Technical Committee. These findings will inform the necessary actions to increase awareness and improve the COVID-19 vaccination strategy among HCWs.

## Supporting information

S1 File Questionnaire. (PDF)

## Abbreviations

HCWs: Healthcare workers
rRT-PCR: real-time reverse-transcriptase polymerase-chain-reaction
WHO: World Health Organization
VOCs: variants of concern
FDA: Food and Drug Administration
ARDS: acute respiratory distress syndrome
PPE: personal protective equipment
IPC: infection prevention and control
LMICs: low- and middle-income countries
CFR: case fatality rate
COVAX: COVID-19 Vaccines Global Access
CDC: Communicable Disease Control
DGHS: Director General of Health Services
US CDC: US Centers for Disease Control and Prevention
IRB: institutional review board
PMT: protection motivation theory
VH: vaccine hesitancy
icddr,b: International Centre for Diarrhoeal Disease Research Bangladesh
DNA: Deoxyribonucleic acid
CT value: cycle threshold
WGS: Whole genome sequencing
ELISA: The enzyme-linked immunosorbent assay
IgG: Immunoglobulin G
OR: Odds ratios
CI: confidence intervals
NITAG: National Immunization Technical Advisory Group
