# Supplementary material for: Study protocol for COVID-19 breakthrough infections and vaccine-induced immune response among a cohort of healthcare workers, Bangladesh
Source: PLoS One. 2024 Dec 31;19(12):e0316121. doi: 10.1371/journal.pone.0316121 (PMC11687639; doi:10.1371/journal.pone.0316121)
Supplement: S1 File — (PDF) [file pone.0316121.s001.pdf]

Title: Health survey among health care workers through establishment of a health care worker cohort in Bangladesh
Baseline Questionnaire
(To be completed by the study physician)

Section A: Eligibility Criteria		
**Inclusion criteria:** All answers must be **YES** for the participant to be enrolled. If any answer is **NO** the participant cannot be enrolled.		
**Sl. no.**	**Questions**	**Category and code**
1	Is the participant a health care worker (All staff in the healthcare facility involved in the provision of care of patients, including those who may not have provided direct care to the patient but who have had contact with the patient's body fluids, potentially contaminated items or environmental surfaces.) who is directly or indirectly involved in patient care and also eligible for COVID-19 vaccination without any contraindication?	☐No=0 ☐Yes=1
2	Is the participant willing to provide written informed consent?	☐No=0 ☐Yes=1
**Exclusion criteria:** All answers must be **NO** for the participant to be enrolled. If any answer is **YES**, the participant cannot be enrolled.		
3	Is the participant a basic medical science faculty or an administrative staff?	☐No=0 ☐Yes=1
4	Is the participant not willing to undergo follow up?	☐No=0 ☐Yes=1
**Confirmation of Eligibility**		
5	Name..	
6	Address..	
7	Phone no.1............................ Phone no.2............................	
8	Name of interviewer..	
	Signature of interviewer	
9	Date	D | D | M | M | Y | Y
10	Participant's ID<br>Please prepare ID as follows: DD|MM|YY|WorkerType|Hospital ID|HCW serial no<br>D | D | M | M | Y | Y | WT | WT | HID | HID | HCW | HCW | HCW | HCW	

Title: Health survey among health care workers through establishment of a health care worker cohort in Bangladesh
Baseline Questionnaire
(To be completed by the study physician)

**Section B: Participant Particulars**		
**Sl. No.**	**Question**	**Category and code**
11	Study site	☐ Dhaka=1 ☐ Chittagong=2 ☐Nilphamari=3 ☐Jessore=4
12	Participant's ID<br>Please prepare ID as follows: DD|MM|YY|WorkerType|Hospital ID|HCW serial **no**<br>D | D | M | M | Y | Y | WT | WT | HID | HID | HCW | HCW | HCW | HCW	
13	Date of Interview	D D M M Y Y
14	Time of Interview	H H : M M , P M
15	Specimen collected	☐No=0 ☐Yes=1
15.1	Serial no. of sample	________
16.1	Symptom status	☐Asymptomatic=0 ☐Symptomatic=1
16.2	Type of Specimen<br>**(Tick all that apply)**	
16.3	Blood/serum	☐No=0 ☐Yes=1
16.4	Pooled throat and nasal swab	☐No=0 ☐Yes=1
17	Date of specimen collection	D D M M Y Y
18	Time of Sample Collection	H H : M M , P M
18a	Result: PCR	☐Negative=0 ☐ Positive=1 ☐Unknown=88
18b	Result: IgG Level	
18c	Result: Variant	

**Section C: Socio-Demography**		
**Sl. No.**	**Question**	**Category and code**
19	Age	☐☐ (In years)☐☐ (In months)
20	Date of Birth	D D M M Y Y
21	Sex	☐ Male=1 ☐ Female=2

Title: Health survey among health care workers through establishment of a health care worker cohort in Bangladesh
Baseline Questionnaire
(To be completed by the study physician)

**Section C: Socio-Demography**		
**Sl. No.**	**Question**	**Category and code**
22	Blood group	
23	Marital Status	☐ Unmarried=1 ☐ Married=2 ☐ Divorced=3<br>☐ Separated=4 ☐ Widow=5 ☐ Widower=6
24	Respondent’s religion	☐ Muslim=1<br>☐ Hindu=2<br>☐ Christian =3<br>☐ Buddhist=4<br>☐ Other=5<br>If others “yes” please specify<br>……………………………………………………
25	Family Size<br>**Note:** Living under the same roof and sharing food cooked in same pot)<br>☐☐ (In round number)	
26	Living status	☐ Single=1<br>☐ Nuclear family=2<br>☐ Joint family=3<br>☐ Mess/dormitory=4<br>☐ Others=5<br>If others “yes” please specify<br>……………………………………………………
27	Participant's residence type	☐ Flat/Apartment=1<br>☐ House/Bungalow=2<br>☐ Tin shed roof=3<br>☐ Others=4<br>If others “yes” please specify<br>……………………………………………………

Title: Health survey among health care workers through establishment of a health care worker cohort in Bangladesh
Baseline Questionnaire
(To be completed by the study physician)

Section C: Socio-Demography		
**Sl. No.**	**Question**	**Category and code**
28	Is the respondent's residence rented, owned?<br>☐Rented=1<br>☐Owned=2	
29	Participant's highest level of education	☐Never went to school=1<br>☐Less than primary=2<br>☐Completed primary=3<br>☐Completed SSC=4<br>☐Completed HSC=5<br>☐Completed Graduation=6<br>☐Completed Post graduation=7
30	Total family income per month in taka	TK……………………………….
31	Total family expenditure per month in taka	TK……………………………...
32	Occupational level in health care facility?<br>☐Medical doctor general ward=1<br>☐Medical doctor in CCU/ICU/emergency/outpatient=2<br>☐ Registered nurse in general ward=3<br>☐Registered nurse in CCU/ICU/emergency/outpatient=4<br>☐Ward boy or cleaner in general ward=5<br>☐Ward boy or cleaner in CCU/ICU/emergency/outpatient=6<br>☐Consultant Radiologist=7<br>☐Radiology technician=8<br>☐Phlebotomist/Laboratory personnel/technician=9<br>☐others=10<br>If other “yes” please specify role:<br>………………………………………………………………………	

Title: Health survey among health care workers through establishment of a health care worker cohort in Bangladesh
Baseline Questionnaire
(To be completed by the study physician)

**Section C: Socio-Demography**		
**Sl. No.**	**Question**	**Category and code**
33	Which type of ward the participant is engaged with?	☐Non-Covid=1<br>☐Covid=2<br>☐Both=3
34	Which department the participant is employed in?	☐Intensive Care Unit (ICU)=1<br>☐Surgery=2<br>☐Medicine=3<br>☐Obstetrics=4<br>☐ Emergency Department=5<br>☐Pediatrics and/or Pediatric Specialties=6<br>☐Gynecology and/or Obstetrics=7<br>☐Oncology and/or Hematology=8<br>☐Dentistry=9<br>☐Radiology=10<br>☐Outpatient clinic=11<br>☐Pharmacy==12<br>☐Laboratory=13<br>☐Nutrition=14<br>☐Social Assistance=15<br>☐Physiotherapy=16<br>☐Occupational therapy=17<br>☐Other=18<br>If other "yes" please specify the department:<br>……………………………………………………………<br>……………………………………………………………
35	How long do you work in ward/department per /week?	

Title: Health survey among health care workers through establishment of a health care worker cohort in Bangladesh
Baseline Questionnaire
(To be completed by the study physician)

**Section C: Socio-Demography**		
**Sl. No.**	**Question**	**Category and code**
	☐☐☐ (In hours)	
36	How long are you in this profession?<br>☐☐(In years)☐☐(In months)	
37	How long are you posted in this hospital?<br>☐☐(In years)☐☐(In months)	
38	How long are you posted in this ward?<br>☐☐(Years)☐☐(Months)	
39.1	History of tobacco usage	☐No=0 ☐Yes=1
39.2	If 'Yes' how many sticks per day?	☐☐(In per days)
**History of past illness /pre-existing co-morbid chronic conditions**		
40.1	Does the participant have any history of past illness or co-existing co-morbid conditions?	☐No=0 ☐Yes=1☐Unknown=88<br>**Skip Note**: If the answer is 'No,' skip to Q.41; if the answer is 'Yes,' specify *as many co-morbid chronic conditions as possible*.
40.2	Cancer	☐No=0 ☐Yes=1☐Unknown=88
40.3	Hypertension	☐No=0 ☐Yes=1☐Unknown=88
40.4	Diabetes	☐No=0 ☐Yes=1☐Unknown=88
40.5	HIV/other immune deficiency	☐No=0 ☐Yes=1☐Unknown=88
40.6	Heart disease	☐No=0 ☐Yes=1☐Unknown=88
40.7	Stroke	☐No=0 ☐Yes=1☐Unknown=88
40.8	Asthma<br>(Requiring medication)	☐No=0 ☐Yes=1☐Unknown=88
40.9	Chronic Lung disease<br>(Non-asthma and requiring medication)	☐No=0 ☐Yes=1☐Unknown=88
40.10	Chronic liver disease	☐No=0 ☐Yes=1☐Unknown=88
40.11	Chronic hematological disorder	☐No=0 ☐Yes=1☐Unknown=88

Title: Health survey among health care workers through establishment of a health care worker cohort in Bangladesh
Baseline Questionnaire
(To be completed by the study physician)

Section C: Socio-Demography		
**Sl. No.**	**Question**	**Category and code**
40.12	Chronic kidney disease	☐No=0 ☐Yes=1☐Unknown=88
40.13	Chronic neurological impairment/disease	☐No=0 ☐Yes=1☐Unknown=88
40.14	Mental illness	☐No=0 ☐Yes=1☐Unknown=88
40.15	Organ or bone marrow recipient	☐No=0 ☐Yes=1☐Unknown=88
40.16	History of any prior surgery	☐No=0 ☐Yes=1☐Unknown=88
40.17	Percutaneous coronary intervention (PCI)	☐No=0 ☐Yes=1☐Unknown=88
40.18	High blood cholesterol	☐No=0 ☐Yes=1☐Unknown=88
40.19	Family history of cardiovascular disease	☐No=0 ☐Yes=1☐Unknown=88
40.20<br><br>40.21	Other pre-existing condition(s)<br><br>If other "yes" please specify	☐No=0 ☐Yes=1☐Unknown=88<br><br>...
41.1<br><br>41.2	Are you currently pregnant?<br><br>If pregnant, specify trimester	☐No=0 ☐Yes=1 ☐Unknown=88<br>**Skip note:** Applicable only for female participant<br>☐ First=1 ☐ Second=2<br>☐ Third=3 ☐ Unknown=88
42	Do you take any of the following medications for chronic health conditions?<br>**Note:** Tick all that apply	☐Anti-hypertensive=1<br>☐Anti-depressant=2<br>☐Anxiolytic=3<br>☐Anti-psychotic=4<br>☐Anti-diabetic=5<br>☐Anti-coagulants=6<br>☐Anti-arrhythmic=7<br>☐Lipid lowering agents=8<br>☐Asthma medication=9<br>☐Steroids=10<br>☐Other=11

Title: Health survey among health care workers through establishment of a health care worker cohort in Bangladesh
Baseline Questionnaire
(To be completed by the study physician)

Section C: Socio-Demography		
**Sl. No.**	**Question**	**Category and code**
		If others "yes" please specify.....................................

Section D: History of COVID-19 and related illness since the beginning of pandemic (March 2020)		
How many times you developed COVID-19 related illness		☐(In round number)
For each covid-19 episodes please use additional page to describe symptoms.		
Episode No ………………………………………………………………☐(mention episode number)		
**Sl. No.**	**Question**	**Category and code**
43	Have you experienced any respiratory symptoms (fever $\geq 38^0$C or history of fever, sore throat, cough, running nose, shortness of breath) in the period since the start of pandemic in March 2020 in Bangladesh?	☐No=0 ☐Yes=1☐Unknown=88<br>**Skip Note**: If the answer is 'No,' skip to Q.68; if the answer is 'Yes,' specify *as many types of symptoms as possible, along with the date and duration of each.*
44	Date of symptom onset	D D M M Y Y
45.1	Type of respiratory symptoms; **Fever**	☐No=0 ☐Yes=1☐Unknown=88
45.2	If 'Yes' date of onset	D D M M Y Y
45.3	Duration of fever	☐☐(In days)
46.1	**Cough**	☐No=0 ☐Yes=1☐Unknown=88
46.2	If 'Yes' date of onset	D D M M Y Y
46.3	Duration of cough	☐☐(In days)
47.1	**Difficulty breathing**	☐No=0 ☐Yes=1☐Unknown=88
47.2	If 'Yes' date of onset	D D M M Y Y
47.3	Duration of difficulty breathing	☐☐(In days)
48.1	**Sore throat**	☐No=0 ☐Yes=1☐Unknown=88
48.2	If 'Yes' date of onset	D D M M Y Y

Title: Health survey among health care workers through establishment of a health care worker cohort in Bangladesh
Baseline Questionnaire
(To be completed by the study physician)

Section D: History of COVID-19 and related illness since the beginning of pandemic (March 2020)		
48.3	Duration of sore throat	☐☐(In days)
49.1	**Runny nose**	☐No=0 ☐Yes=1☐Unknown=88
49.2	If 'Yes' date of onset	D D M M Y Y
49.3	Duration of runny nose	☐☐(In days)
50.1	**Chills**	☐No=0 ☐Yes=1☐Unknown=88
50.2	If 'Yes' date of onset	D D M M Y Y
50.3	Duration of chills	☐☐(In days)
51.1	**Vomiting**	☐No=0 ☐Yes=1☐Unknown=88
51.2	If 'Yes' date of onset	D D M M Y Y
51.3	Duration of vomiting	☐☐(In days)
52.1	**Nausea**	☐No=0 ☐Yes=1☐Unknown=88
52.2	If 'Yes' date of onset	D D M M Y Y
52.3	Duration of nausea	☐☐(In days)
53.1	**Diarrhea**	☐No=0 ☐Yes=1☐Unknown=88
53.2	If 'Yes' date of onset	D D M M Y Y
53.3	Duration of diarrhea	☐☐(In days)
54.1	**Headache**	☐No=0 ☐Yes=1☐Unknown=88
54.2	If 'Yes' date of onset	D D M M Y Y
54.3	Duration of headache	☐☐(In days)
55.1	**Rash**	☐No=0 ☐Yes=1☐Unknown=88
55.2	If 'Yes' date of onset	D D M M Y Y
55.3	Duration of rash	☐☐(In days)
56.1	**Conjunctivitis**	☐No=0 ☐Yes=1☐Unknown=88

**Section D: History of COVID-19 and related illness since the beginning of pandemic (March 2020)**		
56.2	If 'Yes' date of onset	D D M M Y Y
56.3	Duration of conjunctivitis	☐☐ (In days)
57.1	**Muscle aches**	☐No=0 ☐Yes=1☐Unknown=88
57.2	If 'Yes' date of onset	D D M M Y Y
57.3	Duration of muscle aches	☐☐(In days)
58.1	**Joint aches**	☐No=0 ☐Yes=1☐Unknown=88
58.2	If 'Yes' date of onset	D D M M Y Y
58.3	Duration of joint aches	☐☐(In days)
59.1	**Loss of appetite**	☐No=0 ☐Yes=1☐Unknown=88
59.2	If 'Yes' date of onset	D D M M Y Y
59.3	Duration of loss of appetite	☐☐(In days)
60.1	**Loss of smell (Anosmia)**	☐No=0 ☐Yes=1☐Unknown=88
60.2	If 'Yes' date of onset	D D M M Y Y
60.3	Duration of loss of smell	☐☐(In days)
61.1	**Loss of Taste**	☐No=0 ☐Yes=1☐Unknown=88
61.2	If 'Yes' date of onset	D D M M Y Y
61.3	Duration of loss of taste	☐☐(In days)
62.1	**Nose bleed**	☐No=0 ☐Yes=1☐Unknown=88
62.2	If 'Yes' date of onset	D D M M Y Y
62.3	Duration of nose bleed	☐☐(In days)
63.1	**Fatigue**	☐No=0 ☐Yes=1☐Unknown=88
63.2	If 'Yes' date of onset	D D M M Y Y
63.3	Duration of fatigue	☐☐(In days)

Title: Health survey among health care workers through establishment of a health care worker cohort in Bangladesh
Baseline Questionnaire
(To be completed by the study physician)

Section D: History of COVID-19 and related illness since the beginning of pandemic (March 2020)		
64.1	**Seizures**	☐No=0 ☐Yes=1 ☐Unknown=88
64.2	If 'Yes' date of onset	D D M M Y Y
64.3	Duration of seizures	☐☐(In days)
65.1	**Altered consciousness**	☐No=0 ☐Yes=1 ☐Unknown=88
65.2	If 'Yes' date of onset	D D M M Y Y
65.3	Duration of altered consciousness	☐☐(In days)
66.1	**Other neurological signs**	☐No=0 ☐Yes=1 ☐Unknown=88
66.2	If 'Yes' specify	Specify..
66.3	If 'Yes' date of onset	D D M M Y Y
66.4	Duration of other neurological signs	☐☐(In days)
67.1	**Other Symptoms**	☐No=0 ☐Yes=1 ☐Unknown=88
67.2	If 'Yes' specify,	Specify..
67.3	If 'Yes' date of onset	D D M M Y Y
67.4	Duration of onset	☐☐(In days)
67.5	Have you tested for COVID-19 after experiencing respiratory symptoms?	☐No=0 ☐Yes=1
68	Have you ever been diagnosed with COVID-19?	☐No=0 ☐Yes=1<br>**Skip Note**: If the response is 'No,' skip to Q.91; *if the response is 'Yes,' complete Q69-Q90.*
69	Date of Diagnosis	D D M M Y Y
70	If the date is unknown, please specify only the year and month.	☐☐(In years)<br>☐☐(In month)

Title: Health survey among health care workers through establishment of a health care worker cohort in Bangladesh
Baseline Questionnaire
(To be completed by the study physician)

Section D: History of COVID-19 and related illness since the beginning of pandemic (March 2020)		
71	What type of test was done?	☐ PCR=1<br>☐ Rapid Antigen Test=2<br>☐ Serology Test=3<br>☐ I don't remember=4
72	What was the test(s) result(s)?	☐ PCR positive=1<br>☐ PCR negative=2<br>☐ Serology positive=3<br>☐ Serology Negative=4<br>☐ Rapid antigen positive=5<br>☐ Rapid antigen negative=6
73	What symptoms did you experience before being diagnosed with COVID-19?<br>**Note:** Tick all that apply	☐ Fever=1<br>☐ Cough=2<br>☐ Runny nose=3<br>☐ Sore throat=4<br>☐ Difficulty breathing=5<br>☐ Other=6<br>If other "yes" please specify....................................
74<br>75	Did you require hospitalization<br>Duration of hospitalization	☐No=0 ☐Yes=1<br>☐☐(In days)
76	What type of hospital	☐Private=1 ☐Public=2
77	If you were hospitalized, was it for respiratory illness?	☐No=0 ☐Yes=1
78	Did you require oxygen supplementation	☐No=0 ☐Yes=1
79<br>80	Did you require ICU support<br>Duration of ICU	☐No=0 ☐Yes=1<br>☐☐(In days)
81.1	Did you have any complication?	☐No=0 ☐Yes=1

Title: Health survey among health care workers through establishment of a health care worker cohort in Bangladesh
Baseline Questionnaire
(To be completed by the study physician)

Section D: History of COVID-19 and related illness since the beginning of pandemic (March 2020)		
81.2	If others "yes", please specify	…………………………………………………………………………
82	What medications you were treated with for your COVID-19 diagnosis?<br>**Note:** Tick all that apply	☐Antibiotic=1<br>☐Anti-viral=2<br>☐Ivermectin=3<br>☐Hydroxychloroquine=4<br>☐Blood Thinners=5<br>☐Others=6<br>If other "yes" please specify…………………………………
83	What other medical tests were performed for the COVID-19 diagnosis?<br>**Note:** Tick all that apply	☐Chest X-ray=1<br>☐CT Scan=2<br>☐ECG=3<br>☐Echocardiogram=4<br>☐CBC=5<br>☐Markers of blood clotting=6<br>☐Others=7<br>If other "yes" please specify…………………………………
84	Did you get re-tested for COVID-19 (for first positive test)	☐No=0 ☐Yes=1
85	If 'Yes' what was the result of last re-test?	☐Negative=0 ☐ Positive=1
86	If 'Negative' how long it took to get negative after the first COVID-19 diagnosis?	☐☐(In round number)
87	For subsequent infection with COVID-19 (if any), please mention date of onset and which test was done?	D D M M Y Y<br>☐PCR=0 ☐ Rapid antigen=1 ☐ Serology=2
88	Number of your family member/s diagnosed as confirmed COVID-19 case?	☐☐(In round number)
89	Did you experience any symptoms after recovery from COVID-19?	☐No=0 ☐Yes=1

Title: Health survey among health care workers through establishment of a health care worker cohort in Bangladesh
Baseline Questionnaire
(To be completed by the study physician)

**Section D: History of COVID-19 and related illness since the beginning of pandemic (March 2020)**	
90	What are the symptoms you experienced after recovery from COVID-19?<br>1.................................... 2....................................<br>3.................................... 4....................................

Section E: COVID-19 Risk exposure		
Sl. No	Question	Category and Code
91	Were you ever exposed or in contact to a confirmed COVID-19 patient?	☐No=0 ☐Yes=1☐Unknown=88<br>Skip Note: If the answer is 'No', skip to Q.103; if the answer is 'Yes',start providing answer from Q.92…
92	Day or month of last exposure to COVID-19 patient	D D M M Y Y
93	Type of setting where HCW had last exposure to COVID patient<br>Note: Tick all that apply	☐ Inpatient ward=1<br>☐ ICU unit=2<br>☐ CCU unit=3<br>☐ Primary health care=4<br>☐ Home care=5<br>☐ Other=6<br>If other "yes" please specify....................................
If the HCW were exposed to a confirmed COVID patient what were the activities performed on COVID-19 patient in any setting,		
94	Did you provide direct care to a confirmed COVID-19 patient?	☐No=0 ☐Yes=1☐Unknown=88
95.1	Did you have face-to-face contact (within 1 meter) with a confirmed COVID-19 patient in a health care facility?	☐No=0 ☐Yes=1☐Unknown=88
95.2	If 'Yes' did you wear PPE?	☐No=0 ☐Yes=1
95.3	If you wore PPE, what type?<br>Note: Tick all that apply	☐ Medical or surgical mask=1<br>☐ Respirator (ffp2/n95/equivalent) =2<br>☐ Face shield=3

Title: Health survey among health care workers through establishment of a health care worker cohort in Bangladesh
Baseline Questionnaire
(To be completed by the study physician)

Section E: COVID-19 Risk exposure		
Sl. No	Question	Category and Code
		☐ Gloves=4<br>☐ Goggles=5<br>☐ Gown or coverall=6<br>☐ Head cover=7<br>☐ Shoe cover=8
96.1	Was any aerosol-generating procedure performed on the patient *(such as Tracheal intubation/Nebulizer treatment/Open airway suctioning/Collection of sputum/Tracheotomy/Bronchoscopy/Cardiopulmonary resuscitation/Other)?*	☐No=0 ☐Yes=1 ☐Unknown=88
96.2	If 'Yes' what type of procedure	☐Tracheal intubation=1<br>☐Nebulizer treatment=2<br>☐Open airway suctioning=3<br>☐Collection of sputum=4<br>☐Tracheotomy=5<br>☐Bronchoscopy=6<br>☐Cardiopulmonary resuscitation=7<br>☐Other=8<br>If others "yes" please specify....................................
97.1	Were you present when any aerosol-generating procedures were performed on the patient?	☐No=0 ☐Yes=1 ☐Unknown=88
97.2	If 'Yes' did you wear PPE during the procedure?	☐No=0 ☐Yes=1
97.3	If you wore PPE, what type?<br>Note: Tick all that apply	☐ Medical or surgical mask=1<br>☐ Respirator (ffp2/n95/equivalent) =2<br>☐ Face shield=3<br>☐ Gloves=4<br>☐ Goggles=5<br>☐ Gown or coverall=6<br>☐ Head cover=7

Title: Health survey among health care workers through establishment of a health care worker cohort in Bangladesh
Baseline Questionnaire
(To be completed by the study physician)

Section E: COVID-19 Risk exposure		
Sl. No	Question	Category and Code
		☐ Shoe cover=8
98.1	Did you have direct contact with the environment/surfaces where the confirmed COVID-19 patient was cared for? *E.g. bed, linen, medical equipment, bathroom etc.*	☐No=0 ☐Yes=1 ☐Unknown=88
98.2	If yes which materials/surface<br>Note: Tick all that apply<br>☐ Clothes=1<br>☐ Personal items=2<br>☐ Linen=3<br>☐Medical devices used on the patient=4<br>☐Medical equipment connected to the patient (ventilator, infusion pump etc.) =5<br>☐ Bed=6<br>☐ Bathroom=7<br>☐ Ward corridor=8<br>☐ Patient table=9<br>☐ Bedside table=10<br>☐ Dining table=11<br>☐ Medical gas panel=12<br>☐Other=13<br>If others please specify..	
98.3	Did you wear PPE when you came into contact with the patient's environment/surface?	☐No=0 ☐Yes=1 ☐Unknown=88
98.4	If you wore PPE, what type?<br>Note: Tick all that apply	☐ Medical or surgical mask=1<br>☐ Respirator (ffp2/n95/equivalent) =2<br>☐ Face shield=3<br>☐ Gloves=4<br>☐ Goggles=5

Title: Health survey among health care workers through establishment of a health care worker cohort in Bangladesh
Baseline Questionnaire
(To be completed by the study physician)

Section E: COVID-19 Risk exposure		
Sl. No	Question	Category and Code
		☐ Gown or coverall=6<br>☐ Head cover=7<br>☐ Shoe cover=8
99	Did you perform hand hygiene before contact with the patient's materials?	☐No=0 ☐Yes=1☐Unknown=88
If the HCW took care of COVID-19 patient within last 14 days,		
100	Number of times the HCW had close contact (within 1 meter) with admitted COVID-19 patient during last 14 days (in numbers)<br>☐☐(In round number)	
101	What is the maximum duration the health care worker had close contact with admitted COVID-19 patient?	☐<5 minutes=1 ☐5-15 minutes=2<br>☐>15 minutes=3
102.1	Did you come into contact with the patient's body fluids?	☐No=0 ☐Yes=1☐Unknown=88
102.2	If you came into contact with the patient's body fluids, did you wear PPE?	☐No=0 ☐Yes=1☐Unknown=88
102.3	If you wore PPE, what type?<br>Note: Tick all that apply	☐ Medical or surgical mask=1<br>☐ Respirator (ffp2/n95/equivalent)=2<br>☐ Face shield=3<br>☐ Gloves=4<br>☐ Goggles=5<br>☐ Gown or coverall=6<br>☐ Head cover=7<br>☐ Shoe cover=8
103	In the past 14 days, how often have you used public transport?	☐ Most days (≥ 8 days)=1<br>☐ Some days (4–7 days)=2<br>☐Few days (≤ 3 days)=3<br>☐Not used public transport=4

Title: Health survey among health care workers through establishment of a health care worker cohort in Bangladesh
Baseline Questionnaire
(To be completed by the study physician)

Section E: COVID-19 Risk exposure		
Sl. No	Question	Category and Code
103.1	How often have you used a mask while using public transport?	☐ Always=1<br>☐ Often =2<br>☐ Sometimes=3<br>☐ Rarely=4<br>☐ Never=5
104	In the past 14 days, how often have you had social interaction (e.g. close contact, touching, talking etc.) with individuals outside of work, at home or transport (e.g. in markets, shops etc.)	☐ Most days (≥ 8 days)=1<br>☐ Some days (4–7 days)=2<br>☐ Few days (≤ 3 days)=3<br>☐ Not had any other social interaction=4
104.1	How often have you worn a mask when in an indoor setting outside of your home?	☐ Always=1<br>☐ Often =2<br>☐ Sometimes=3<br>☐ Rarely=4<br>☐ Never=5

**Section F: Vaccination History**								
**Sl. No**	**Question**	**Category and Code**						
105.1	Did you receive **COVID-19** vaccine?	☐ No=0 ☐ Yes=1						
105.2	a. If 'Yes' which dose	☐ 1st dose=1<br>☐ 2nd dose=2<br>☐ 3rd dose (Booster)=3<br>☐ 4th dose (Booster)=4						
105.3	b. Date of vaccination	First dose	D	D	M	M	Y	Y
		Second dose	D	D	M	M	Y	Y
		3rd dose (Booster)	D	D	M	M	Y	Y
		4th dose (Booster)	D	D	M	M	Y	Y
105.4	c. Which vaccine did you receive?	1st dose						
		2nd dose						

Title: Health survey among health care workers through establishment of a health care worker cohort in Bangladesh
Baseline Questionnaire
(To be completed by the study physician)

**Section F: Vaccination History**									
**Sl. No**	**Question**	**Category and Code**							
		3rd dose (Booster)							
		4rd dose (Booster)							
105.5	d. If 'No' why not	Specify..							
106.1	Do you have a contraindication for the COVID-19 vaccine?	☐No=0 ☐Yes=1 ☐Unknown=88							
106.2	If yes, specify	Specify..							
106.3	Side effects after vaccine uptake	Ist Dose	2nd Dose	3rd Dose	4th Dose				
		☐ Injection site pain<br>☐ Fever<br>☐ Fatigue<br>☐ Headache<br>☐ Myalgia<br>☐ Other<br>___________	☐ Injection site pain<br>☐ Fever<br>☐ Fatigue<br>☐ Headache<br>☐ Myalgia<br>☐ Other<br>___________	☐ Injection site pain<br>☐ Fever<br>☐ Fatigue<br>☐ Headache<br>☐ Myalgia<br>☐ Other<br>___________	☐ Injection site pain<br>☐ Fever<br>☐ Fatigue<br>☐ Headache<br>☐ Myalgia<br>☐ Other<br>___________				
**Other vaccines**									
107.1	Have you received an **influenza** vaccine in the current influenza season?	☐No=0 ☐Yes=1							
107.2	If yes, when did you receive the influenza vaccine			D	D	M	M	Y	Y
107.3	Name of influenza vaccine received (product name)	☐Vaxigrip=1<br>☐Influvax=2<br>☐ Fluarix=3<br>☐Agrippal S1=4							
107.4	If 'No' why not	Specify..							
108.1	Has the participant received **Hepatitis-B** vaccine?	☐No=0 ☐Yes=1							
108.2	If 'Yes' Date of vaccination	1st dose		D	D	M	M	Y	Y
108.3		2nd dose		D	D	M	M	Y	Y
108.4		3rd dose		D	D	M	M	Y	Y

Title: Health survey among health care workers through establishment of a health care worker cohort in Bangladesh
Baseline Questionnaire
(To be completed by the study physician)

Section F: Vaccination History		
**Sl. No**	**Question**	**Category and Code**
108.5	Type of vaccination	☐Engerix-B=1<br>☐Euvax-B=2<br>☐Hepa-B=3<br>☐Hepavax-B=4
108.6	If 'No' why not	Specify...

Section G: Acceptance of COVID-19 booster vaccine		
**Attitude towards the COVID-19 booster vaccine:**		
1	"When a coronavirus booster vaccination becomes available to you, are you going to take one?"	☐No=0 ☐Yes=1 ☐Not Sure=3
	What are the reasons you are willing to receive the booster shot of the COVID-19 vaccine? [multiple choice]	☐It is safe to vaccinate with the booster vaccine.<br>☐The booster vaccine is effective.<br>☐The emergence of the mutant virus will reduce the protective effect of the previous vaccination.<br>☐Suggestions or recommendations from others.<br>☐Others.
2	Please specify why you are not willing or not sure to take the vaccine? (multiple responses can be given)	☐I am afraid of the needle<br>☐I am not sure if the vaccine will be free or not<br>☐The vaccine might have side effects/safety concerns<br>☐I don't know for how many days I can get protection by getting the vaccine<br>☐I have doubts about the proper preservation of the vaccine<br>☐For religious beliefs/reasons<br>☐I don't know where to get the vaccine<br>☐I don't know anything about the vac- cine<br>☐Others. (Please specify):
**PMT (Protection Motivation Theory) Scale**		

Title: Health survey among health care workers through establishment of a health care worker cohort in Bangladesh
Baseline Questionnaire
(To be completed by the study physician)

1	**If you infected with COVID-19, it will seriously damage your health.**	☐Strongly disagree<br>☐Disagree<br>☐Neither agree nor disagree<br>☐Agree<br>☐Strongly agree
2	**If you infected with COVID-19, it is a great possibility of long-term sequelae.**	☐Strongly disagree<br>☐Disagree<br>☐Neither agree nor disagree<br>☐Agree<br>☐Strongly agree
3	**If you infected with COVID-19, It will seriously affect the health of family members.**	☐Strongly disagree<br>☐Disagree<br>☐Neither agree nor disagree<br>☐Agree<br>☐Strongly agree
4	**You are likely to be infected with COVID-19 in the next 12 months.**	☐Strongly disagree<br>☐Disagree<br>☐Neither agree nor disagree<br>☐Agree<br>☐Strongly agree
5	**People around you are likely to be infected with COVID-19 in the next 12 months**	☐Strongly disagree<br>☐Disagree<br>☐Neither agree nor disagree<br>☐Agree<br>☐Strongly agree
6	**Taking the booster vaccine can help you prevent COVID-19 effectively.**	☐Strongly disagree<br>☐Disagree<br>☐Neither agree nor disagree<br>☐Agree<br>☐Strongly agree
7	**Taking the booster vaccine is beneficial to your work and life during the Pandemic.**	☐Strongly disagree<br>☐Disagree<br>☐Neither agree nor disagree<br>☐Agree<br>☐Strongly agree
8	**Taking the booster vaccine is beneficial to your family and members of society.**	☐Strongly disagree<br>☐Disagree<br>☐Neither agree nor disagree<br>☐Agree<br>☐Strongly agree

**9**	**It is convenient to you to take the booster vaccine.**	☐Strongly disagree<br>☐Disagree<br>☐Neither agree nor disagree<br>☐Agree<br>☐Strongly agree
**10**	**It is important to you to take the booster vaccine.**	☐Strongly disagree<br>☐Disagree<br>☐Neither agree nor disagree<br>☐Agree<br>☐Strongly agree
**11**	**You will receive the COVID-19 vaccine according to the recommendations of the disease control department or professional doctors.**	☐Strongly disagree<br>☐Disagree<br>☐Neither agree nor disagree<br>☐Agree<br>☐Strongly agree
**12**	**There will be side effects after booster vaccination.**	☐Strongly disagree<br>☐Disagree<br>☐Neither agree nor disagree<br>☐Agree<br>☐Strongly agree
**13**	**It will have a negative impact on my future health after booster vaccination.**	☐Strongly disagree<br>☐Disagree<br>☐Neither agree nor disagree<br>☐Agree<br>☐Strongly agree
**VH (Vaccine Hesitancy) Scale**		
**1**	**The COVID-19 booster vaccine is effective.**	☐Strongly disagree<br>☐Disagree<br>☐Neither agree nor disagree<br>☐Agree<br>☐Strongly agree
**2**	**It's important to get the booster vaccine to strengthen my health.**	☐Strongly disagree<br>☐Disagree<br>☐Neither agree nor disagree<br>☐Agree<br>☐Strongly agree
**3**	**It is important for the health of community residents to be vaccinated with COVID-19 booster vaccine.**	☐Strongly disagree<br>☐Disagree<br>☐Neither agree nor disagree<br>☐Agree

Title: Health survey among health care workers through establishment of a health care worker cohort in Bangladesh
Baseline Questionnaire
(To be completed by the study physician)

		☐Strongly agree
**4**	**The information I received about the booster vaccine is reliable.**	☐Strongly disagree<br>☐Disagree<br>☐Neither agree nor disagree<br>☐Agree<br>☐Strongly agree
**5**	**Booster Vaccination is a good way to protect people around my family (friends, family members) from COVID-19 infection.**	☐Strongly disagree<br>☐Disagree<br>☐Neither agree nor disagree<br>☐Agree<br>☐Strongly agree
**6**	**I am worried about the possible side effects of a COVID-19 booster vaccine.**	☐Strongly disagree<br>☐Disagree<br>☐Neither agree nor disagree<br>☐Agree<br>☐Strongly agree
**7**	**There is more risk of booster vaccination than that of ordinary shot.**	☐Strongly disagree<br>☐Disagree<br>☐Neither agree nor disagree<br>☐Agree<br>☐Strongly agree
**8**	**If the COVID-19 booster vaccine is recommended by the government, I believe booster vaccination is beneficial.**<br>**The recommendation for the COVID-19 booster vaccine by doctors, community and other professionals has a great influence on me**	☐Strongly disagree<br>☐Disagree<br>☐Neither agree nor disagree<br>☐Agree<br>☐Strongly agree
**9**	**In the current situation, booster vaccination may not be necessary.**	☐Strongly disagree<br>☐Disagree<br>☐Neither agree nor disagree<br>☐Agree<br>☐Strongly agree

**Section H: Physical examination**		
109	Weight *in Kg*	☐☐

Title: Health survey among health care workers through establishment of a health care worker cohort in Bangladesh
Baseline Questionnaire
(To be completed by the study physician)

**Section H: Physical examination**		
110	Height *in cm*	☐☐☐
111	Waist Circumference *in cm*	☐☐
112	Hip circumference *in cm*	☐☐
113	Systolic Blood Pressure*inmm of Hg*	☐☐☐
114	Diastolic Blood pressure*inmm of Hg*	☐☐☐
115	Blood Glucose level *(mmol/l)*	☐.☐☐
116	Lipid profile:	
	Cholesterol	☐. ☐☐
	TG	☐. ☐☐
	HDL	☐. ☐☐
	LDL	☐. ☐☐
	Cholesterol/HDL cholesterol	☐. ☐☐
